# A Multicenter Retrospective Study of Frameless Robotic Radiosurgery for Intracranial Arteriovenous Malformation

**DOI:** 10.3389/fonc.2014.00298

**Published:** 2014-11-04

**Authors:** Eric K. Oermann, Nikhil Murthy, Viola Chen, Advaith Baimeedi, Deanna Sasaki-Adams, Kevin McGrail, Sean P. Collins, Matthew G. Ewend, Brian T. Collins

**Affiliations:** ^1^Department of Neurological Surgery, Icahn School of Medicine at Mount Sinai, New York, NY, USA; ^2^Department of Neurological Surgery, Georgetown University School of Medicine, Washington, DC, USA; ^3^Department of Radiation Medicine, Georgetown University School of Medicine, Washington, DC, USA; ^4^Department of Neurological Surgery, The University of North Carolina at Chapel Hill, Chapel Hill, NC, USA

**Keywords:** stereotactic radiosurgery, arteriovenous malformations, image guided radiation therapy, outcomes, CT angiography

## Abstract

**Introduction:** CT-guided, frameless radiosurgery is an alternative treatment to traditional catheter-angiography targeted, frame-based methods for intracranial arteriovenous malformations (AVMs). Despite the widespread use of frameless radiosurgery for treating intracranial tumors, its use for treating AVM is not-well described.

**Methods:** Patients who completed a course of single fraction radiosurgery at The University of North Carolina or Georgetown University between 4/1/2005–4/1/2011 with single fraction radiosurgery and received at least one follow-up imaging study were included. All patients received pre-treatment planning with CTA ± MRA and were treated on the CyberKnife (Accuray) radiosurgery system. Patients were evaluated for changes in clinical symptoms and radiographic changes evaluated with MRI/MRA and catheter-angiography.

**Results:** Twenty-six patients, 15 male and 11 female, were included in the present study at a median age of 41 years old. The Spetzler-Martin grades of the AVMs included seven Grade I, 12 Grade II, six Grade III, and one Grade IV with 14 (54%) of the patients having a pre-treatment hemorrhage. Median AVM nidal volume was 1.62 cm^3^ (0.57–8.26 cm^3^) and was treated with a median dose of 1900 cGy to the 80% isodose line. At median follow-up of 25 months, 15 patients had a complete closure of their AVM, 6 patients had a partial closure, and 5 patients were stable. Time since treatment was a significant predictor of response, with patients experience complete closure having on average 11 months more follow-up than patients with partial or no closure (*p* = 0.03). One patient experienced a post-treatment hemorrhage at 22 months.

**Conclusion:** Frameless radiosurgery can be targeted with non-invasive MRI/MRA and CTA imaging. Despite the difficulty of treating AVM without catheter angiography, early results with frameless, CT-guided radiosurgery suggest that it can achieve similar results to frame-based methods at these time points.

## Introduction

Intracranial arteriovenous malformations (AVMs) present one of the greatest clinical challenges for neurosurgeons, radiation oncologists, and neurointerventionalists. Classically, the treatment of these lesions involved careful patient selection followed by large, open surgical procedures, or more recently endovascular obliteration, radiosurgery, or a combination of these methods (1–3). This trend of utilizing increasingly less invasive options, endovascular, and radiosurgical, has lead to the advent of frameless radiosurgical devices that do not require the traditional head frame for stereotaxic guidance (4, 5). Despite the widespread adoption of these devices for treating both intracranial and extracranial pathologies, to the author’s knowledge to date there has been only two reports on the results of frameless radiosurgical devices for the treatment of intracranial AVM (5, 6). We report the retrospective results of two institutions with using CyberKnife frameless stereotactic radiosurgery (SRS) for the treatment of intracranial AVM.

## Methods

### Patient selection and treatment

**Figure 1:**
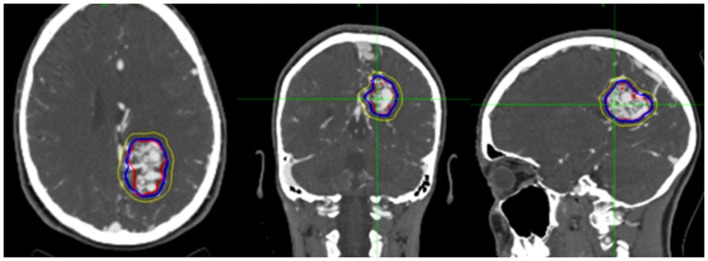
**Figure demonstrating treatment planning for a representative case**. Planned treatment volume (red), 90% isodose (blue), and 50% isodose (yellow) can be seen in three planes on pre-treatment planning CTA.

We performed a retrospective review of patients with intracranial AVMs treated with CyberKnife SRS from December 1st, 2005 to February 1st, 2011 at Georgetown University Hospital and the University of North Carolina at Chapel Hill. Patients who had undergone single fraction SRS for intracranial AVM with or without endovascular embolization and had received at least one follow-up imaging study were included. All patients were treated by an interdisciplinary team of radiation oncologists and neurosurgeons. High resolution CTA images with or without MRA were obtained from all patients for pre-treatment planning. A planning target volume (PTV) and critical structures were manually delineated by the treating neurosurgeon with the PTV encompassing the contour of the AVM with a 1 mm margin (Figure 1). All treatment planning was performed on pre-treatment CTA imaging, and when available, using fused MRA/CTA imaging. The treating isodose and prescription dose were determined by the treating radiation oncologist in consultation with the treating neurosurgeon, and took into account the AVM nidus, overall volume, proximity to critical structures, and previous treatment history. Treatment plans were generated using an inverse planning method by the CyberKnife treatment software (Multiplan, Accuray).

### Outcomes assessment

Patients were tracked as part of routine clinical follow-up by the interdisciplinary team. MRA scans with or without catheter-angiography confirmation were obtained at pre-defined annual intervals unless acute changes in neurological status warranted immediate imaging. Neurological symptoms were clinically assessed and recorded by the treating neurosurgeons. Complete closure was defined as total resolution of the AVM nidus and draining veins on imaging, with partial closure being defined as a decrease in size of the nidus with the persistence of large draining veins.

### Statistical analysis

All statistical analyses were performed utilizing SPSS Statistics v19 (IBM). Statistical analysis was performed in order to identify pre-treatment and treatment variables that correlated with AVM closure. The Kruskal–Wallis test, a non-parametric equivalent to ANOVA, was utilized for comparison of continuous variables grouped by AVM closure outcomes. For analysis of volume and dose, Pearson Chi-square testing was employed. Alpha was set to 0.05 to yield a 95% confidence interval (CI) for all statistical tests. Averages were all reported as the median value and interquartile range (IQR), which is a more robust measure of dispersion than simple range.

## Results

### Patient and treatment characteristics

Twenty-six patients were identified as having undergone treatment for intracranial AVM and met all criteria for inclusion in the current study (Table 1). Fifteen (58%) of the patients were male and 11 (42%) were female. The median age at time of treatment with radiosurgery was 41 years (IQR, 26–55 years). The AVMs had a range of Spetzler–Martin grades with 7 Grade I, 12 Grade II, 6 Grade III, and 1 Grade IV. Ten (38%) of the patients were either current smokers or had a history of smoking, and seven (23%) of the patients were hypertensive. Fourteen (54%) of the patients had a pre-treatment hemorrhage, and of the hypertensive patients, six out of seven (86%) experienced pre-treatment hemorrhage (*p* = 0.027). The median AVM nidus volume was 1.62 cm^3^ (IQR, 0.57–8.26 cm^3^). Pre-treatment embolization was performed in 11 patients (42%), with 9 patients being treated with Onyx and the others with *n*-butyl cyanoacrylate (NBCA). The median isodose was 80% (76–83%), which was treated with a median prescription dose of 1900 cGy (IQR, 1800-2175 cGy). Seventeen (65%) of the patients had SRS as monotherapy, while nine underwent a combination of SRS and embolization or, in one case, surgical resection.

**Table 1:** **Summary of AVM patient characteristics**.

Variable	Value
Subjects, *N*	26
Median age, years (IQR)	41 (26–55)
Gender
Male, *n* (%)	15 (58)
Female, *n* (%)	11 (42)
Smoking status
Current/prior history, *n* (%)	10 (38)
Never, *n* (%)	12 (46)
Unknown, *n* (%)	4 (15)
Pre-treatment neurological symptoms
Headache, *n* (%)	12 (46)
Seizures, controlled/uncontrolled, *n*/*n* (%/%)	2/2 (8/8)
Motor deficits, *n* (%)	9 (35)
Pre-treatment hemorrhage, *n* (%)	14 (54)
Hypertension (%)	7 (27)
Pre-treatment hemorrhage, *n* (%)	6 (86)
No pre-treatment hemorrhage, *n* (%)	1 (14)
Spetzler martin grade
I, *n* (%)	7
II, *n* (%)	12
III, *n* (%)	6
IV, *n* (%)	1
Median Nidus volume, cm^3^ (IQR)	1.62 (0.57–8.26)
Intervention
SRS only, *n* (%)	17 (65)
SRS + embolization or surgery, *n* (%)	9 (35)
Isodose, median % (IQR)	80 (76–83%)
Dose, median cGy (IQR)	1900 (1800–2175)

### AVM Closure rates

**Figure 2:**
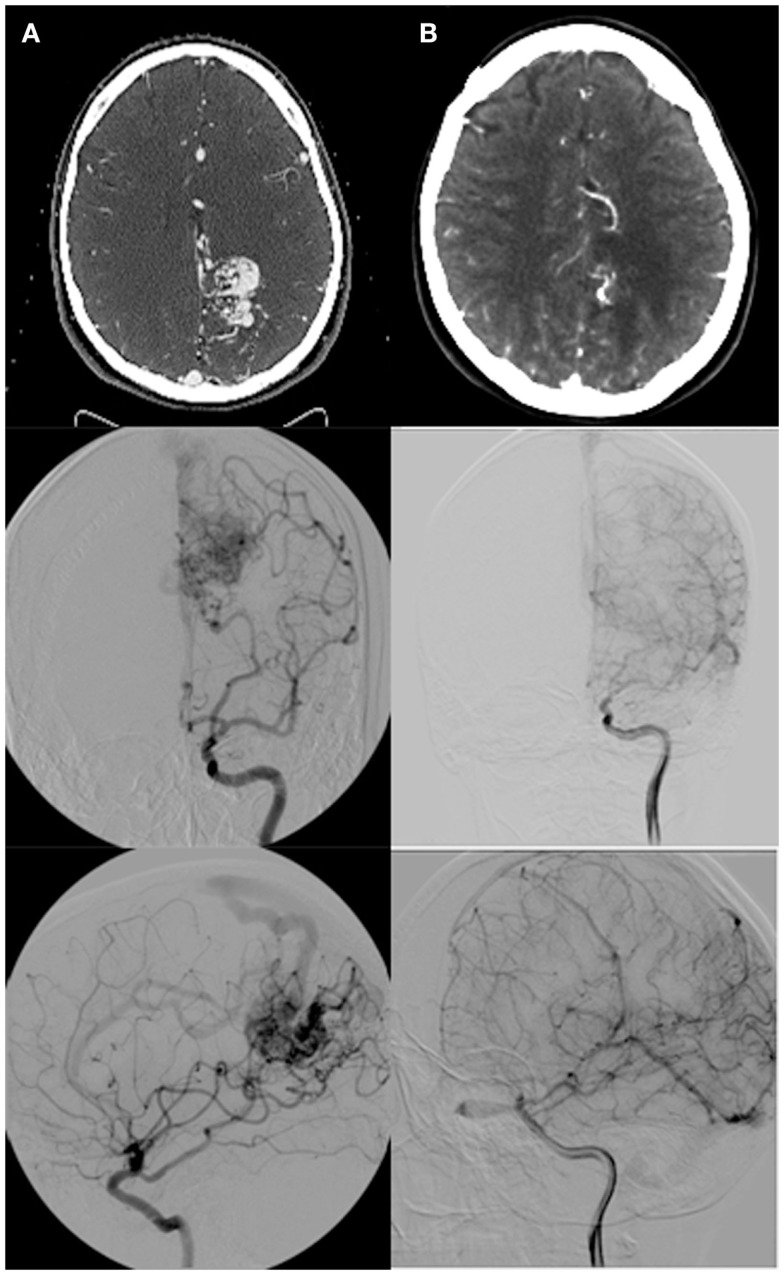
**(A)** Pre-radiosurgery, and **(B)** post-radiosurgery CT angios and angiograms for a representative case with follow-up images taken at 2 years post-radiosurgery demonstrating complete nidal obliteration with no residual draining vein.

At median follow-up for the cohort of 25 months (IQR, 19-36 months), 15 patients had a complete closure of their AVM, 6 patients had a partial closure, and 5 patients were stable (Figure 2). Time since treatment was a significant predictor of response, fully closed AVM had, on average, 11 months more follow-up time than those with partial or no closure (*p* = 0.03) (Table 2). Nidal volume and dose did not correlate with AVM closure rate (*p* = 0.63, 0.12). Spetzler–Martin Grade did not correlate with AVM closure as well (*p* = 0.26).

**Table 2:** **Summary of AVM treatment characteristics and patient outcomes**.

Variable	Endpoint			(*p*-Value)
	Complete closure (*n* = 15)	Partial closure (*n* = 6)	Stable (*n* = 5)
Median follow-up, months (IQR)	31 (24–39)	17 (11–22)	25 (18–29)	0.03
Spetzler martin grade	–	–	–	0.26
I, *n* (%)	5 (33)	3 (50)	0 (0)
II, *n* (%)	8 (53)	3 (50)	2 (40)
III, *n* (%)	3 (20)	0 (0)	3 (60)
IV, *n* (%)	1 (6)	0 (0)	0 (0)
Median Nidus volume, cm^3^ (IQR)	1.15 (0.54–4.66)	3.42 (0.71–10.24)	4.42 (1.96–9.07)	0.63
Intervention				0.64
SRS only, *n* (%)	8 (53)	5 (83)	4 (80)
SRS + embolization or surgery, *n* (%)	7 (47)	1 (17)	1 (20)
Isodose, median % (IQR)	80 (76–85%)	77 (72–80%)	81 (80–81%)	0.24
Dose, median Gy (IQR)	1800 (1750–1950)	2050 (2000–2175)	2000 (2000–2200)	0.12

### Neurological deficits and toxicity

One patient experienced a post-treatment hemorrhage at 22 months requiring emergent surgical decompression (Table 3). No other significant post-treatment adverse events were reported. The most common pre-treatment neurological symptom was headaches (46%), which improved in most cases after treatment with only four patients (15%) reporting them at the end of the study. Pre-treatment, controlled, and uncontrolled seizures were symptoms in 16% of the patients. By conclusion of the study, 12% of the patients had controlled seizures on oral medications, and no patients had uncontrolled seizures.

**Table 3:** **Summary of post-treatment adverse events and symptoms**.

Variable	Value
Post-treatment hemorrhage, *n* (%)	1 (4)
Post-treatment neurological symptoms
Headache, *n* (%)	4 (15)
Seizures, controlled/uncontrolled, *n*/*n* (%/%)	3/0 (12/0)
Motor deficits, *n* (%)	3 (12)

## Discussion

Our results show that frameless SRS is a safe and effective technique for the treatment of intracranial AVM. A large recent study by Ding et al. reported an obliteration rate of 30% at 10 years, and the present study with an obliteration rate of 58% at 3 years compares favorably to these results (7). The higher rate of closure in the present study is may be due to generally smaller nidal volumes, and generally lower grades, yet roughly equivalent marginal doses (7). It is worth noting that Spetzler–Martin grading incorporates size into its calculation of grade (as well as draining veins and eloquence of cortex), and therefore is unsurprisingly correlated with obliteration rates. The results of this article with regards to obliteration rates and dependent factors are consistent with observations made in similar studies of radiosurgical outcomes for treatment of intracranial AVM with Gamma Knife (7–10). While other studies have shown a consistent and expected dependence of AVM closure on dose, volume, grade, and follow-up time, the present study only demonstrated a dependence on follow-up time (8). This lack of dependence upon dose and volume may be attributable to a small sample size and the variance within these factors, and therefore are negative results due to lack of statistical power rather than truly negative results.

Our minimally invasive approach of obtaining CTA with or without MRA for planning purposes prior to frameless SRS does come with a notable drawback when treating AVM after embolization with Onyx. Onyx, an ethylene vinyl alcohol polymer which is solvated in dimethyl-sulfoxide (DMSO), is radio-opaque and can cause artifact on CTA, which can make it difficult to properly visualize the AVM nidus for treatment planning and follow-up.

The distribution of post-treatment neurological complications in the present group compared similarly to reported series within the Gamma Knife literature as well, with a significant improvement occurring for major neurological symptoms including seizures and motor function compared to pre-operative symptoms (9, 11). For pre-treatment headaches, there was 66% rate of total resolution, identical to the results of Steiner et al. (11).

Approximately, half of the patients in the present series experienced pre-treatment hemorrhage. Pre-treatment hemorrhage can vary greatly between studies in the literature, with some cohorts consisting almost entirely of patients with hemorrhage, and others entirely without (7, 11). Recent studies have shown that post-radiosurgery hemorrhage can increase the time until AVM closure, and previous work by Flickinger demonstrated that pre-treatment hemorrhage can have a lasting impact on the resolution of neurologic sequelae, although this last finding has been disputed (7, 12, 13).

## Conclusion

This small pilot series demonstrates that frameless SRS is a safe and effective measure for treating intracranial AVM in utilizing the traditional single fraction approach. Due to advanced imaging and motion tracking technologies, it can achieve equivalent results to traditional frame-based methods without the need for pins and a stereotaxic frame. With further research, we may be able to maximize the benefits of this novel technology for the treatment of intracranial AVM.

## Conflict of Interest Statement

Brian T. Collins and Sean P. Collins have received honoraria from Accuray Inc. for previous work as consultants. The other co-authors declare that the research was conducted in the absence of any commercial or financial relationships that could be construed as a potential conflict of interest.
